# Supplementary material for: Implementation and validation of a new qPCR assay to detect imported human Plasmodium species
Source: Microbiol Spectr. 2024 Dec 10;13(1):e01622-24. doi: 10.1128/spectrum.01622-24 (PMC11705816; doi:10.1128/spectrum.01622-24)

**Supplemental data**

**Supplemental Tables**

**Supplemental Table 1. Limit of detection of the pan-*Plasmodium* qPCR assay using a control-plasmid.**

| **DNA copies/µL** | **Cq 1** | **Cq 2** | **Cq 3** | **Cq 4** | **Cq 5** | **Cq 6** | **Cq 7** | **Cq 8** | **Cq 9** | **Cq 10** | **Mean Cq** | **SD Cq** |
| --- | --- | --- | --- | --- | --- | --- | --- | --- | --- | --- | --- | --- |
| **1000** | 33.76 | 33.86 | 34.2 | 34.09 |  |  |  |  |  |  | 33.98 | 0.20 |
| **100** | 36.67 | 36.98 | 37.27 | 37.06 |  |  |  |  |  |  | 36.99 | 0.25 |
| **10** | 40.01 | 40.81 | 40.55 | 40.29 | 41.01 | 41 | 40.83 | 40.72 | 40.03 | 40.85 | 40.61 | 0.36 |
| **1** | 45 | 42.77 | 45 | 45 | 45 | 45 | 42.67 | 40.92 | 44.22 | 43.84 |  |  |
| **0.1** | Negative | Negative | 45 | Negative | Negative | Negative | 45 | 45 | Negative | Negative |  |  |

Cq, quantification cycle.

**Supplemental Table 2. qPCR efficiency for each qPCR assay.**

| **Species** | **Efficiency** |
| --- | --- |
| *Plasmodium* spp. | 1.70 (85%) |
| *P. falciparum* | 1.87 (93.5%) |
| *P. vivax* | 1.93 (96.5%) |
| *P. ovale* | 1.61 (80.5%) |
| *P. malariae* | 1.68 (84%) |
| *P. knowlesi* | 1.85 (92.5%) |

**Supplemental Table 3. Basic characteristics of 410 patients.**

| **Characteristic** | **Negative BS** | **Positive BS** |
| --- | --- | --- |
| No. of patients | 323 | 87 |
| Malaria species identification, No. (%)  *P. falciparum*  *P. vivax*  *P. ovale*  *P. malariae*  Mixed *Plasmodium* species infection* |  | 59 (67.8)  7 (8)  10 (11.5)  3 (3.5)  8 (9.2) |
| No. of specimens  Day of diagnosis  Follow-up | 333 | 190  87  103 |
| No. of specimens per species, No. (%)  *P. falciparum*  *P. vivax*  *P. ovale*  *P. malariae*  Mixed *Plasmodium* species infection |  | 117 (61.6)  19 (10)  25 (13.2)  9 (4.7)  20 (10.5) |
| No. of specimens per patient, median [range] | 1 [1 – 3] | 2 [1 – 7] |
| Sex ratio (M/F) | 1.03 | 3.35 |
| Age (median) | 41 | 40 |

*Diagnosis of mixed *Plasmodium* species infection was performed by conventional methods (BS or ICT), by qPCR of French National Reference Center of malaria or by our qPCR assays.

**Supplemental Table 4. Comparison of Cq at the single species malaria diagnosis between pan-*Plasmodium* and specific-species qPCRs.**

| **Malaria species identification** | **Target qPCR** | **Cq, median at the diagnosis [IQR]** | **Median parasitaemia at diagnosis (parasites/µL) [IQR]** |
| --- | --- | --- | --- |
| *P. falciparum* | *Plasmodium* spp. | 26.7 [24.6 – 29.6] | 13,350 [4,005 – 46,725] |
|  | *P. falciparum* | 24.2 [22.6 – 27.2] |  |
| *P. vivax* | *Plasmodium* spp. | 28.1 [26.6 – 28.5] | 8,900 [2,336 – 17,800] |
|  | *P. vivax* | 27.9 [26.7 – 28.2] |  |
| *P. ovale* | *Plasmodium* spp. | 29.4 [28 – 32.1] | 2,670 [445 – 4,450] |
|  | *P. ovale* | 36 [35.1 – 38.4] |  |
| *P. malariae* | *Plasmodium* spp. | 29.1 [28.7 – 30] | 5,785 [5,118 – 7,343] |
|  | *P. malariae* | 36.9 [36.2 – 37.3] |  |

Cq, quantification cycle.

**Supplemental Figures**

**Supplemental Figure 1. Alignment of 18S rRNA of the five species of malaria parasites infecting humans (*P. falciparum*, *P. vivax*, *P. ovale*, *P. malariae* and *P. knowlesi*) with other *Apicomplexa* species for primers and probes design.**

(a) Overview of rRNA 18S fragment with two identified regions for qPCR design. (b) “Region 1” used for *Plasmodium* spp., *P. ovale* and *P. malariae* qPCRs with a common reverse primer and probe (FAM). (c) “Region 2” used for *P. falciparum*, *P. vivax* and *P. knowlesi* qPCRs with a common forward primer and probe (HEX).


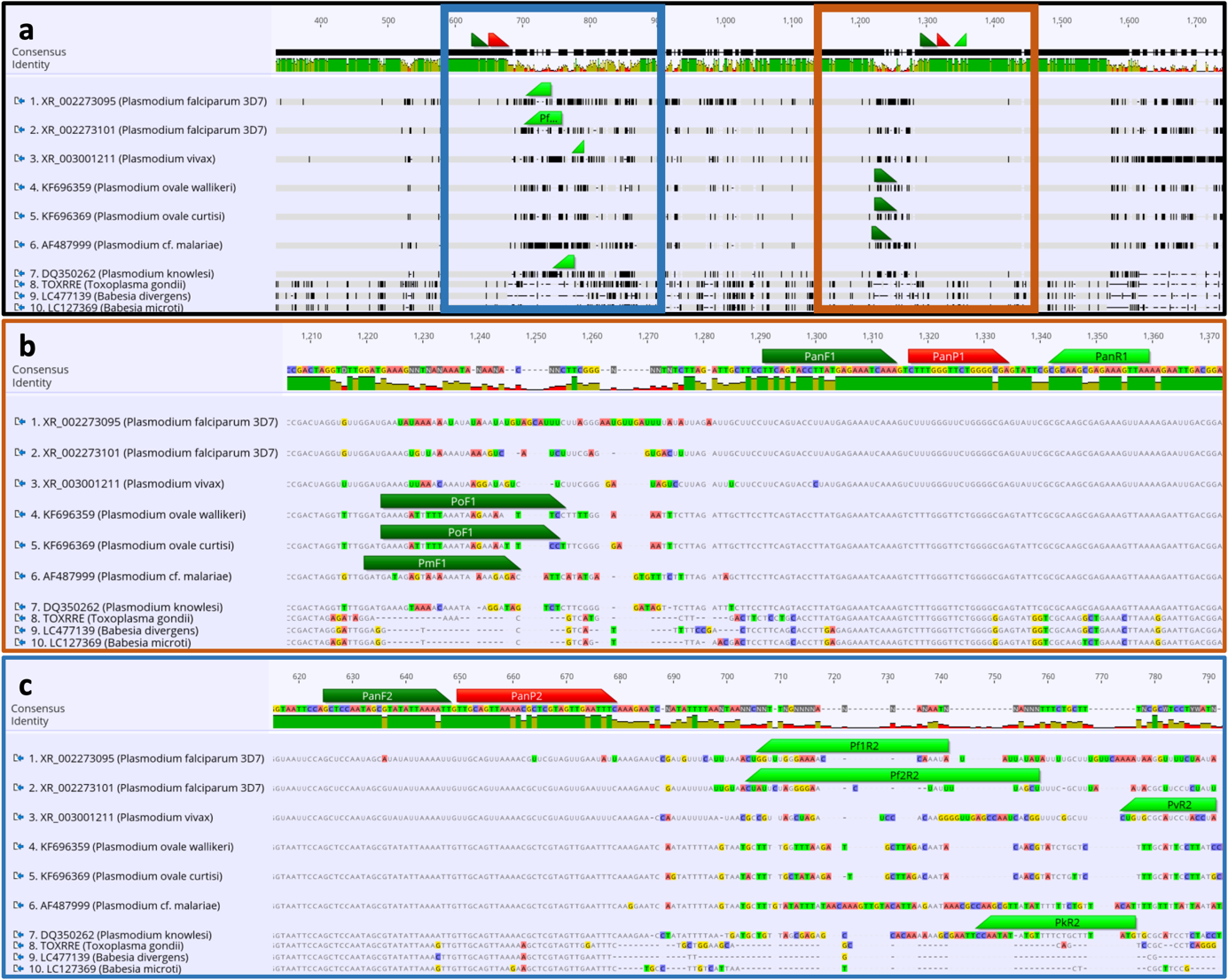


**Supplemental Figure 2. Flowchart of development and validation of qPCR.**

38 clinical samples positive for *P. falciparum*, *P. vivax*, *P. ovale*, *P. malariae* and one culture of *P. knowlesi* were used for qPCRs development. To validate our qPCRs, we used 190 samples from patients diagnosed with malaria (87 samples on the day of diagnosis and 103 samples during post-treatment follow-up) and 333 samples from negative patients by microscopic examination.


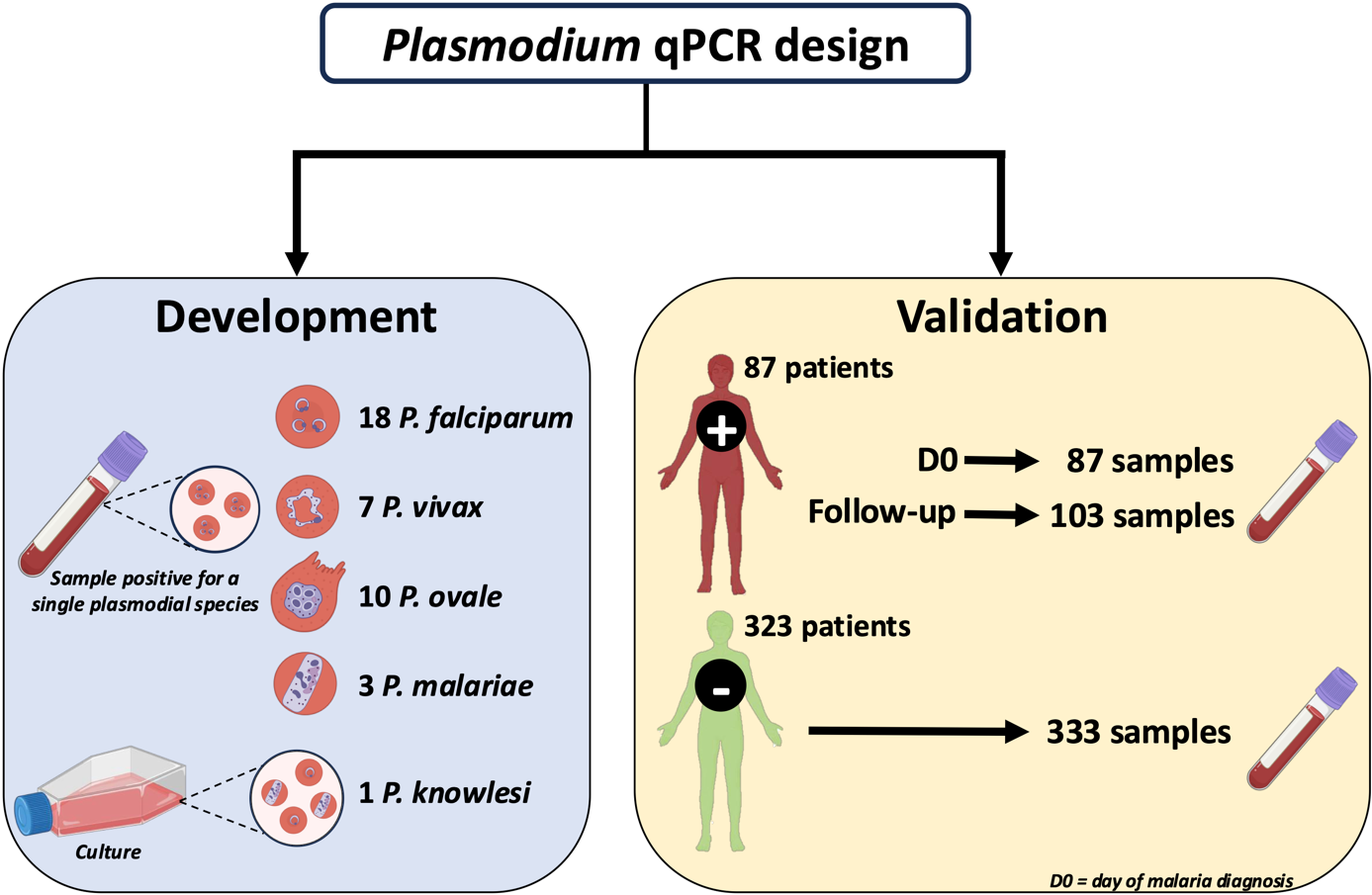


**Supplemental Figure 3. Persistence of circulating *P. vivax*, *P. ovale* and *P. malariae* DNA during post-treatment follow-up with pan-*Plasmodium* qPCR.**

(a) Boxplot represent median (black line) and interquartile range (IQR = Q3 - Q1) between 25^th^ percentile (Q1) and 75^th^ percentile (Q3) of the plasmodial copy number decrease as a function of post-therapy follow-up. Black points represent each value of plasmodial copy number, and large grey point represent the plasmodial copy number average. (b) Connected scatterplot represent the plasmodial copy number decrease for each patient.


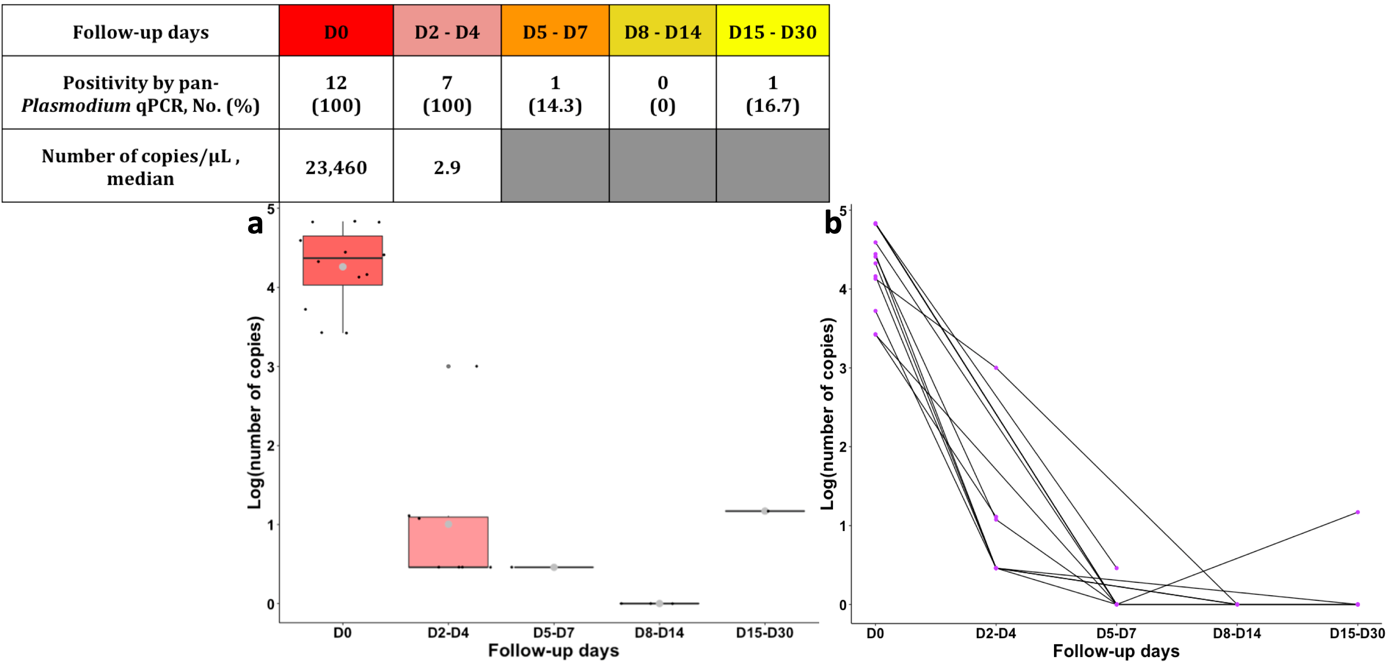

Supplement: Supplemental tables and figures — Tables S1 to S4; Fig. S1 to S3. [file spectrum.01622-24-s0001.docx]
